# An Atypical Presentation of Culture-Negative Cryptococcal Meningitis in an Immunocompetent Host With Prior Stroke: A Case Report

**DOI:** 10.1155/crdi/3055869

**Published:** 2025-11-06

**Authors:** Julia Siau Fang Ting, Arvind Yerramilli, Fiona Clarke, Sarah Wong, Marjoree Sehu

**Affiliations:** ^1^Department of Infectious Diseases, Peninsula Health, Frankston, Victoria, Australia; ^2^Department of Infectious Diseases, Monash Health, Clayton, Victoria, Australia; ^3^Department of Microbiology, Melbourne Pathology, Collingwood, Victoria, Australia

**Keywords:** cryptococcal, immunocompetent, meningitis

## Abstract

Cryptococcal meningitis commonly presents with acute symptoms such as fever and signs of raised intracranial pressure and usually occurs in immunocompromised hosts. A 55-year-old woman presented with worsening cognitive decline, gait disturbance and recurrent falls. Magnetic resonance imaging (MRI) of the brain revealed a prior stroke and ventriculomegaly. A lumbar puncture (LP) showed normal opening pressure. Cerebrospinal fluid (CSF) analysis revealed pleocytosis with raised protein and reduced glucose. Cryptococcal antigen in the CSF was positive; however, *Cryptococcus* was not cultured. The immunodeficiency screen was unremarkable. She was treated with intravenous liposomal amphotericin B and oral flucytosine induction, followed by oral fluconazole consolidation and maintenance. Her cognition and mobility improved markedly after treatment. This case illustrates an uncommon presentation of cryptococcal meningitis and highlights the need to consider this diagnosis in atypical presentations. Culture negativity complicates the determination of optimal treatment duration. This case also highlights the utility of the lateral flow assay (LFA) in diagnosing cryptococcal meningitis.

## 1. Introduction

Cryptococcosis is a fungal infection caused by *Cryptococcus* species. It is an environmental yeast, particularly associated with pigeon droppings, soil and some trees [1]. Infection predominantly occurs through inhalation of spores, with *Cryptococcus neoformans* and *Cryptococcus gattii* being the most clinically significant species [1]. While *C. neoformans* usually affects immunocompromised individuals, *C. gattii* is more commonly seen in immunocompetent hosts [2]. Cryptococcal infection most commonly presents as meningitis, pulmonary or disseminated cryptococcosis [1].

Cryptococcal infection is uncommon in Australia and New Zealand, with a retrospective study conducted across 46 hospitals over a 5-year period identifying 475 cases of cryptococcosis [3].

Here, we present a unique case of an immunocompetent female patient who presented with signs and symptoms suggestive of normal pressure hydrocephalus (NPH) but was unexpectedly diagnosed with cryptococcal meningitis. The diagnosis was confirmed by the detection of cryptococcal antigen (CrAg) in her cerebrospinal fluid (CSF). This case highlights several important diagnostic and therapeutic challenges. It underscores the importance of maintaining a broad differential diagnosis and considering cryptococcal meningitis in patients with ventriculomegaly, even in the absence of typical clinical signs and risk factors.

## 2. Case Presentation

A 55-year-old woman presented with worsening cognitive decline and gait issues, resulting in falls over the past six months. Her past medical history was significant for spontaneous clearance of hepatitis C and a left middle cerebral artery (MCA) infarct in the context of a patent foramen ovale (PFO). This was complicated by residual dysphasia, right-sided weakness, cognitive impairment and reduced functional capacity requiring assistance with personal activities of daily living and mobility. The patient had a history of intravenous drug use, heavy smoking and alcohol misuse. Her regular medications included amitriptyline, pregabalin and Suboxone for chronic pain and prior opioid dependence.

Initially, the patient was enrolled in a home-based rehabilitation programme with regular physiotherapy input. Examination by her primary treating physician showed right upper limb power of 4/5, right hip 3/5 and right knee and ankle 4/5. On the left side, proximal upper and lower limb strength was 4+/5 with distal strength 5/5. She ambulated unsteadily with a 4-wheeled walker. Based on these findings, the initial impression was that of possible NPH, hence was referred to the neurology inpatient team.

On hospital admission, clinical examination by the neurology team revealed mild weakness (power 4/5) and hypertonia in the right upper and lower limbs. Power was normal in the left upper and lower limbs. Bradykinesia was noted during hand opening and closing, along with pronounced right-sided clonus and a withdrawal plantar reflex bilaterally. Sensory and coordination assessments were difficult to complete due to inattention. Reflexes in bilateral upper and lower limbs were normal. The patient had mild dysphasia and demonstrated minimal engagement in conversation with the medical team. Ophthalmoscopy could not be completed due to an inability to follow commands. Cranial nerve examination was incomplete due to inattention; however, there was no obvious facial droop and extraocular movements appeared intact. The physiotherapist's assessment on the day after admission noted that the patient had a wide-based, unsteady gait and required close supervision during mobilisation.

**Figure 1:**
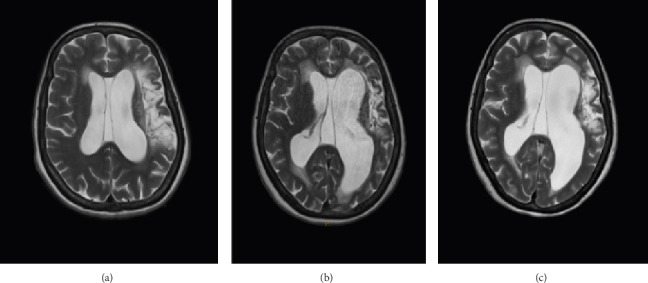
(a) As a comparison, an MRI of the brain 3 years prior to admission showed an established large left MCA territory infarct and hydrocephalus ex vacuo phenomena of the left lateral ventricle. (b) MRI of the brain performed approximately 1.5 months prior to admission showed significant encephalomalacia and gliosis involving the left frontal lobe, resulting in ex vacuo dilatation of the left lateral ventricle and asymmetric ventriculomegaly. (c) A follow-up MRI of the brain, conducted 2 weeks after admission—approximately 2 months after the previous scan (Figure 1(b))—demonstrated persistent chronic compensated hydrocephalus with an extensive left MCA territory infarct, without evidence of meningoencephalitis or cryptococcomas.

Admission blood tests were unremarkable, including inflammatory markers. She achieved a score of 13 out of 36 on the Cognitive Assessment in Stroke Patients (CASP). Magnetic resonance imaging (MRI) of the brain, ordered by the referring physician 1.5 months ago (Figure 1(b)), showed significant encephalomalacia and gliosis involving the left frontal lobe, resulting in ex vacuo dilatation of the left lateral ventricle and asymmetric ventriculomegaly.

Given her clinical presentation and radiology findings, NPH was suspected, and a lumbar puncture (LP) was performed. This revealed a normal CSF opening pressure of 18 cm H_2_O and a white blood cell count of 131 × 10^6^/L (reference range: < 5 × 10^6^/L) with glucose of 0.6 mmol/L (reference range: 2.0–3.9 mmol/L) and protein of 2.41 g/L (reference range: 0.15–0.45 g/L) (Table 1). A subsequent Biofire Filmarray CSF Meningitis/Encephalitis (ME) panel (bioMérieux, Marcy-l'Étoile, France)—which includes testing *for C. neoformans and C. gattii*—along with an acid-fast bacilli (AFB) smear, returned negative results. CSF CrAg testing using the IMMY CrAg lateral flow assay (LFA) (IMMY, Norman, OK, USA) was negative on this first sample, including with additional dilutions to exclude the postzone effect. Serum CrAg was negative.

A computed tomography (CT) scan of the chest, abdomen and pelvis was undertaken to investigate for tuberculosis (TB) or an alternative infective focus. No significant abnormalities were identified, apart from nonspecific changes in the chest. Immunodeficiency tests including white blood cell count, QuantiFERON-TB Gold, immunoglobulins, complements, flow cytometry and HIV were unremarkable.

The patient underwent a repeat LP which again revealed similar biochemistry results with a white blood cell count of 140 × 10^6^/L, glucose of less than 0.6 mmol/L and protein of 1.77 g/L (Table 1). On this sample, TB polymerase chain reaction (PCR) using the Xpert MTB/RIF Ultra assay (Cepheid, Sunnyvale, CA, USA) and initial AFB smear was negative but CSF CrAg was positive with a titre of 1:5. She was started on intravenous liposomal amphotericin B (4 mg/kg, 250 mg daily) and oral flucytosine (25 mg/kg, 1.5 g 6 hourly) as induction therapy.

One week after treatment for cryptococcal meningitis started, the patient's neurological condition significantly improved, as she became more interactive and alert. Physiotherapy assessments indicated that she had returned to her prior level of mobility with a 4-wheeled walker.

A repeat MRI of the brain 2 weeks after admission (Figure 1(c)) showed persistent chronic compensated hydrocephalus with an extensive left MCA territory infarct, with no signs of meningoencephalitis or cryptococcomas. Follow-up LPs showed normal opening pressures, persistent CrAg positivity and improvement in the CSF's glucose and protein levels (Table 1). Panfungal cultures and dedicated cryptococcal PCR testing on the second, third and fourth CSF samples—which were outsourced to the Institute of Clinical Pathology and Medical Research (ICPMR) at Westmead Hospital—returned negative results (Table 1). Mycobacterial cultures processed via the BD BACTEC MGIT automated mycobacterial detection system (BD, Franklin Lakes, NJ, USA) were negative at 6 weeks for both initial LPs.

Following 4 weeks of induction therapy and in the setting of a severe renal impairment, the treatment was changed to oral fluconazole 400 mg daily (renally adjusted from 800 mg daily). Follow-up revealed significant improvement in mobility and cognitive function as reflected by serial CASP testing with a higher score of 22 out of 36. Her LP approximately 11 months after commencement of therapy (at week 47 of admission) revealed a negative CSF CrAg and a marked biochemical improvement (Table 1). After 8 months of fluconazole 400 mg daily, this was reduced to 200 mg daily with the treatment ongoing in the outpatient setting.

### 2.1. Investigations

Summary of the investigations is provided in Table 1.

### 2.2. Imaging

MRI images are shown in Figure 1.

## 3. Discussion

This case report describes an exceptionally atypical presentation of cryptococcal infection in a nonimmunocompromised patient who lacked typical risk factors. While cryptococcal meningitis has been reported in nonimmunocompromised individuals, these occurrences are rare and sparsely documented [4–7].

Cryptococcal meningitis typically presents with an acute onset of fever, headache, altered mental status, nausea and cranial nerve deficits [8]. Altered mental status can be subtle and challenging to recognise in patients with preexisting cognitive impairment, as was the case with our patient. She also did not exhibit classic signs of infection, such as fever and elevated inflammatory markers. The normal opening pressure observed in this patient's LP is unusual, as cryptococcal meningitis typically presents with raised intracranial pressure, often necessitating serial therapeutic LPs [8]. This case underscores the importance of considering cryptococcal infection in patients with isolated ventriculomegaly and progressive cognitive decline.

CSF culture is one of the cornerstones of diagnosing cryptococcal meningitis [2]. However, culture may have been negative in this case due to a low fungal load in the CSF. Immunocompromised hosts typically have a higher fungal burden than nonimmunocompromised hosts [9]. CrAg testing using LFA is increasingly accepted as a diagnostic tool for cryptococcal meningitis due to its more rapid turnover [2]. IMMY CrAg LFA is widely used globally and has been in use for over a decade, demonstrating reliability across various settings [10].

A systematic review and meta-analysis assessing the diagnostic accuracy of CrAg detection for cryptococcal meningitis in adults with HIV found that nearly all included studies employed the IMMY CrAg LFA, except for one that used the StrongStep assay [11]. In serum samples, based on data from three diagnostic cohorts involving 1690 participants, the LFA showed a pooled sensitivity of 97.9% (95% confidence interval [CI]: 87.9–100) and a specificity of 89.5% (95% Cl: 74.3–98.5) (Table 2) [11]. For CSF samples, across six diagnostic cohorts comprising 3099 participants, the pooled sensitivity and specificity were both 99.5% (95% CI: 97.2–99.9 for sensitivity; 94.2–100 for specificity) (Table 2) [11].

Comparable high diagnostic accuracy has been observed in HIV-negative individuals. A systematic review conducted across three continents showed that the IMMY CrAg LFA, used to diagnose cryptococcosis, had a pooled median sensitivity and pooled specificity estimate of 96% for serum samples, with their respective 95% credible interval (CrI) detailed in Table 2 [12]. For CSF samples, the LFA test demonstrated even higher diagnostic performance in this population, with both pooled median sensitivity and specificity at 99% (95% CrI 95%–100%) (Table 2) [12].

Culture-negative cryptococcal cases have been reported in a study where 40% of HIV-associated cryptococcal meningitis patients had positive CSF cryptococcal antigens but sterile cultures [13]. Had the CSF not been tested for cryptococcal antigen in this case, the infection might have gone undetected.

In light of the low pretest probability, the potential for a false-positive cryptococcal antigen result was carefully considered. Nonetheless, this scenario was considered unlikely given the high specificity of the test [11, 12] and the persistently positive cryptococcal antigen in the patient's CSF. The patient also had a dramatic and sustained response to antifungal therapy as evidenced by improvement in neurological status with increased cognitive assessment scores, improved mobility and significantly more engagement with her family, carers and the medical team.

While *C. gattii* and *C. neoformans* are treated with the same antifungal agents—typically amphotericin B and flucytosine followed by fluconazole—*C. gattii* infections often require a longer amphotericin B induction phase of 4–6 weeks, particularly in non-HIV-associated meningitis [2]. *C. gattii* is also more commonly associated with cryptococcomas, especially in the lungs and central nervous system [2]. These mass lesions can lead to neurological symptoms and raised intracranial pressure, necessitating prompt intervention [2]. In such cases, adjunctive treatment with corticosteroids or surgical resection may be considered, making the management of *C. gattii* generally more intensive than that of *C. neoformans* [2].

A few limitations are acknowledged in this case report. The absence of a cultured fungal organism limited species identification and made it difficult to determine the optimal duration of antifungal therapy. There is unclear evidence in guidelines regarding the optimal duration of treatment for culture-negative cryptococcal meningitis as achieving CSF sterility often helps guide treatment decisions during induction [2]. Furthermore, since the patient is not immunocompromised, shorter induction may be suitable, requiring careful consideration of clinical response and risks such as renal impairment in this patient. Another limitation was the patient's baseline dysphasia, which made it challenging to reliably assess her perspective and cognitive improvement. While serial CASP testing showed objective gains, it is uncertain whether this reflected true cognitive recovery or greater engagement due to improved mood. Nevertheless, her family and carers observed meaningful clinical improvement, particularly in mobility and daily interactions.

## 4. Conclusion

Cryptococcal meningitis should be considered in patients presenting with ventriculomegaly and chronic neurological symptoms, even in the absence of classical risk factors. CSF findings of low glucose, elevated protein and lymphocytic pleocytosis should raise clinical suspicion. Importantly, immunocompetent individuals may present with atypical features and negative cultures. Cryptococcal antigen testing is both highly sensitive and specific, and although diagnostic, it does not reliably determine treatment duration.

## Figures and Tables

**Table 1:** Summary of investigations of serial CSF.

Week of admission	CrAg titre	Cryptococcal PCR	Panfungal culture	Protein (g/L) (ref: 0.15–0.45)	Glucose (mmol/L) (ref: 2.0–3.9)	White blood cell count: (ref: < 5 × 10^6^/L)	Polymorphs/mononuclear (× 10^6^/L)	Opening pressure (cmH_2_O)
Week 1	Not detected	Not done	Not done	2.41	0.6	131	90/41	18
Week 2	1:5	Not detected	Not detected	1.77	< 0.6	140	87/53	Not done
Week 3	1:10	Not detected	Not detected	2.45	0.9	144	66/78	15
Week 4	1:10	Not detected	Invalid result^∗^	1.31	1.4	27	1/26	18
Week 5	1:40	Not done	Not done	1.61	1.7	76	12/64	13
Week 47	Not detected	Not done	Not done	0.71	2.9	6	0/6	Not done

*Note:* CrAg, cryptococcal antigen; ref, reference range.

Abbreviation: PCR, polymerase chain reaction.

^∗^The result was invalid as the internal control was not detected.

**Table 2:** Diagnostic performance of IMMY CrAg LFA for cryptococcal infection in HIV-positive and HIV-negative patients [11, 12].

Population	Sample type	IMMY CrAg LFA sensitivity	IMMY CrAg LFA specificity
HIV positive [11]	Serum	97.9% (95% CI: 87.9–100)	89.5% (95% CI: 74.3–98.5)
	CSF	99.5% (95% CI: 97.2–99.9)	99.5% (95% CI: 94.2–100)

HIV negative [12]	Serum	96% (95% CrI: 68%–100%)	96% (95% CrI: 84%–100%)
	CSF	99% (95% CrI: 95%–100%)	99% (95% CrI: 95%–100%)

*Note:* CrAg, cryptococcal antigen; CrI, credible interval.

Abbreviations: CI, confidence interval; LFA, lateral flow assay.

## Data Availability

The data that support the findings of this study are openly available in Zenodo at https://doi.org/10.5281/zenodo.17196417, reference number 10.5281/zenodo.17196417.

## Patient Perspective

The patient's perspective was provided by the carer, as the patient has dysphasia and difficulty expressing herself.

‘I feel that the patient has improved a lot in terms of her alertness and mobility. She is steadier on her feet'.
